# Orchiectomy due to delayed severe scrotal hematocele after laparoscopic transabdominal preperitoneal repair for a giant inguinoscrotal hernia: a case report

**DOI:** 10.1186/s40792-022-01579-3

**Published:** 2022-12-27

**Authors:** Kenichi Nakamura, Susumu Shibasaki, Masashi Takenaka, Akiko Serizawa, Shingo Akimoto, Masaya Nakauchi, Tsuyoshi Tanaka, Kazuki Inaba, Ryoichi Shiroki, Ichiro Uyama, Koichi Suda

**Affiliations:** 1 Department of Surgery, Fujita Health University, 1-98 Dengakugakubo, Kutsukake, Toyoake, Aichi 470-1192 Japan; 2 Department of Urology, Fujita Health University, 1-98 Dengakugakubo, Kutsukake, Toyoake, Aichi 470-1192 Japan; 3 Advanced Robotic and Endoscopic Surgery, Fujita Health University, 1-98 Dengakugakubo, Kutsukake, Toyoake, Aichi 470-1192 Japan; 4 Collaborative Laboratory for Research and Development in Advanced Surgical Technology, Fujita Health University, 1-98 Dengakugakubo, Kutsukake, Toyoake, Aichi 470-1192 Japan; 5 Collaborative Laboratory for Research and Development in Advanced Surgical Intelligence, Fujita Health University, 1-98 Dengakugakubo, Kutsukake, Toyoake, Aichi 470-1192 Japan

**Keywords:** Hernia repair, Postoperative complications, Inguinal hernia

## Abstract

**Background:**

A giant inguinoscrotal hernia is a rare inguinal hernia that extends below the midpoint of the inner thigh while standing. Although reports of laparoscopic surgery for giant inguinoscrotal hernias have increased, the risk of delayed hematocele has not yet been clarified.

**Case presentation:**

A 68-year-old man was evaluated for a left giant inguinoscrotal hernia, and laparoscopic transabdominal preperitoneal repair (TAPP) was performed. In the procedure, the distal hernia sac was not resected. The postoperative course was uneventful for 3 months postsurgery, after which he complained of giant scrotal swelling, which gradually grew to 13 cm. It did not improve with several punctures and caused dysuria because of increased pressure on the urethra. Thus, reoperation was performed 9 months after surgery. The hematocele consisted of a thickened hernia sac, which was tightly adhered to the spermatic cord and testicle. The hernia sac including the hematocele was removed from the scrotum through an anterior approach, preserving the spermatic cord and testicle. On the third postoperative day, an orchiectomy was performed due to poor testicular perfusion caused by spermatic cord injury. There was no hematocele or hernia at the 3-year follow-up. The remnant sac after laparoscopic TAPP for a giant inguinoscrotal hernia possibly caused refractory hematocele. Additionally, the removal of the hernia sac, including hematocele, from the spermatic cord and testicle has a risk of inducing injury, leading to orchiectomy.

**Conclusion:**

Surgeons should be aware of the possibility of delayed refractory hematoceles after laparoscopic TAPP for giant inguinoscrotal hernias when the hernia sac is not resected.

## Background

A giant inguinoscrotal hernia (GISH) is a rare inguinal hernia that extends below the midpoint of the inner thigh while standing [1, 2]. For large inguinoscrotal hernias, the Lichtenstein method is not strongly recommended by the European Hernia Society (EHS) guidelines in 2009 [3]. However, the efficacy to prevent recurrence via the anterior approach for GISH has not been yet established. On the other hand, the International Endohernia Society guidelines (IEHS) weakly recommended performing laparoscopic transabdominal preperitoneal repair (TAPP) as a possible therapeutic option in inguinoscrotal hernias [4], and reports of laparoscopic surgery for GISHs have increased with the advances in laparoscopic procedures and surgical devices [2, 5–7]. However, the general agreement on performing laparoscopic surgery for GISH has not yet been established. In this study, we report a delayed severe hematocele after laparoscopic TAPP for a GISH that led to orchiectomy.

## Case presentation

**Fig. 1:**
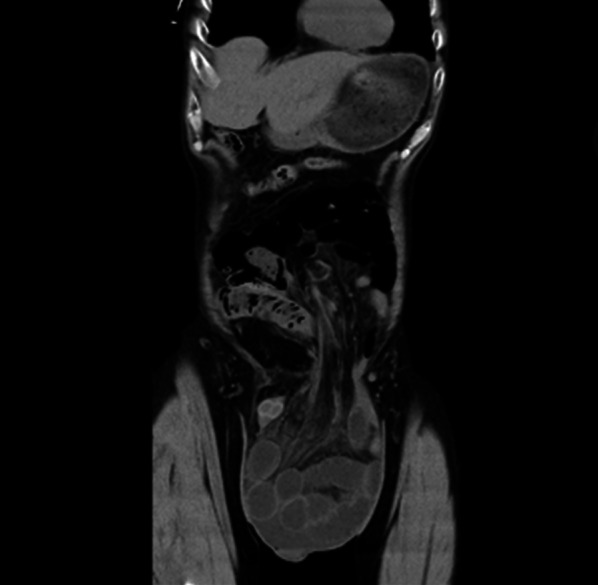
Computed tomography finding of a giant inguinoscrotal hernia

**Fig. 2:**
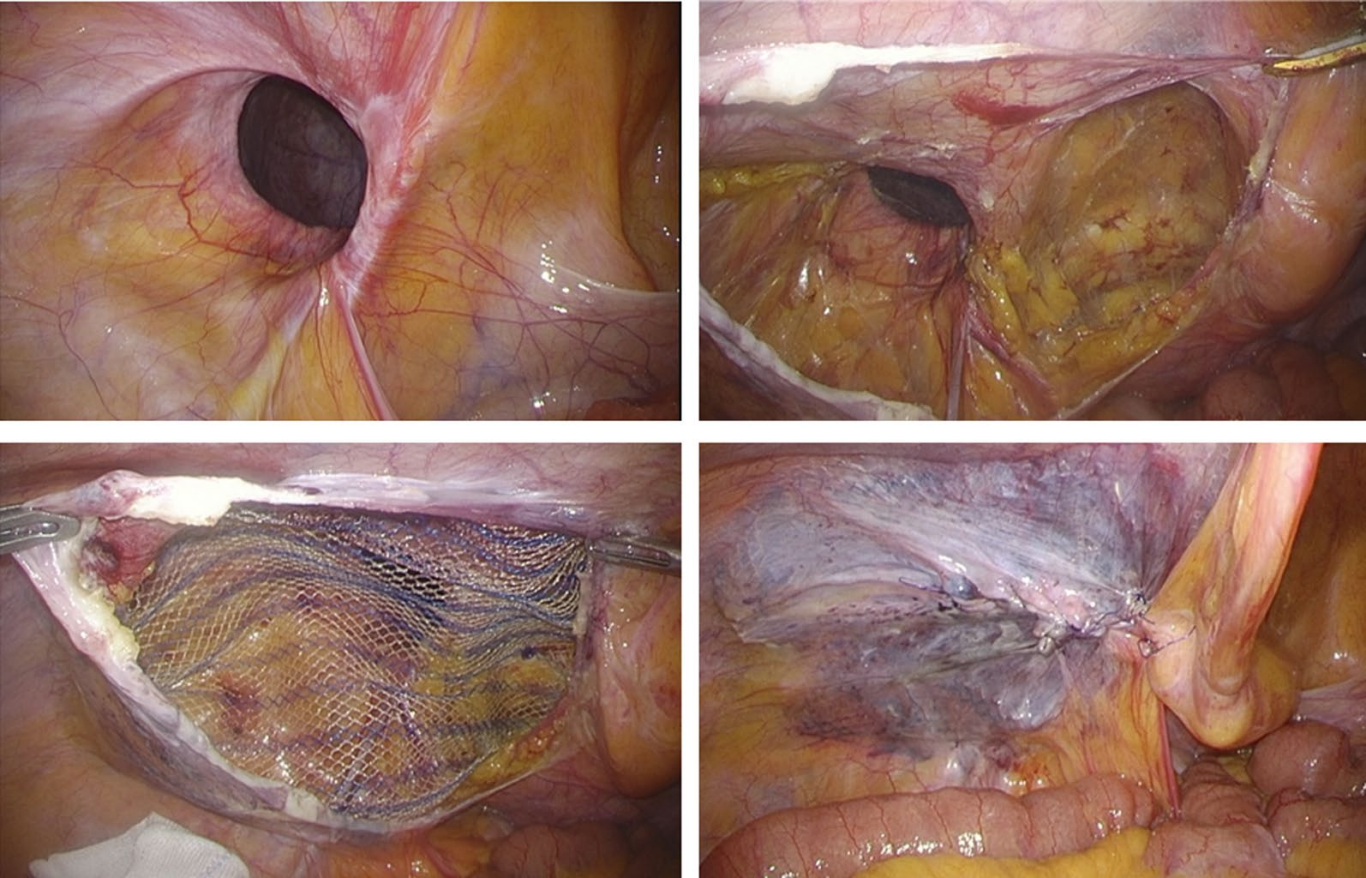
Intraoperative findings during the first operation (laparoscopic transabdominal preperitoneal repair). Blood loss during the operation was minimal

**Fig. 3:**
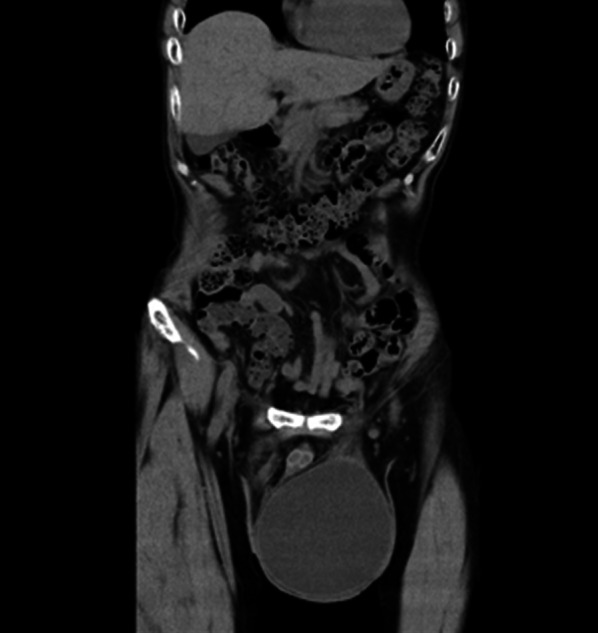
Computed tomography finding of a postoperative giant scrotal fluid collection (hematocele). Pressure on the urethra due to hematocele is observed

A 68-year-old man was evaluated for an incarcerated left GISH (Fig. 1) and laparoscopic TAPP was performed (Fig. 2). The hernia sac was transected circumferentially at the level of the internal inguinal ring. The myopectineal orifice was covered with a prosthetic mesh with absorbable tackers. The peritoneum was closed with continuous sutures (Fig. 2). Operative time was 116 min with a blood loss of 2 ml. Postoperative evolution was uneventful and the patient was discharged on postoperative day (POD) 2. No scrotal swelling was observed during the first 3 months postsurgery, after which he complained of left scrotal swelling, which gradually increased to about 10 cm in diameter 7 months after the operation. It did not improve with several punctures, and caused dysuria due to increased pressure on the urethra (Fig. 3). The patient was reoperated 9 months after the first surgery.

**Fig. 4:**
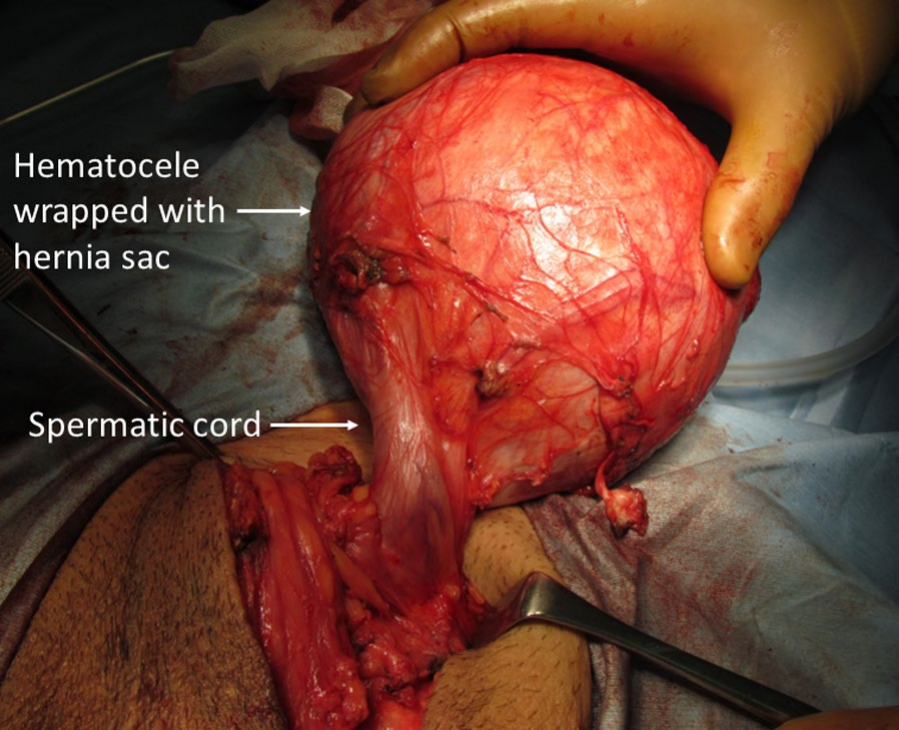
Intraoperative findings during the second operation. A giant hematocele 13 cm in diameter wrapped with a hernia sac was tightly adherent to the spermatic cord and testicles

**Fig. 5:**
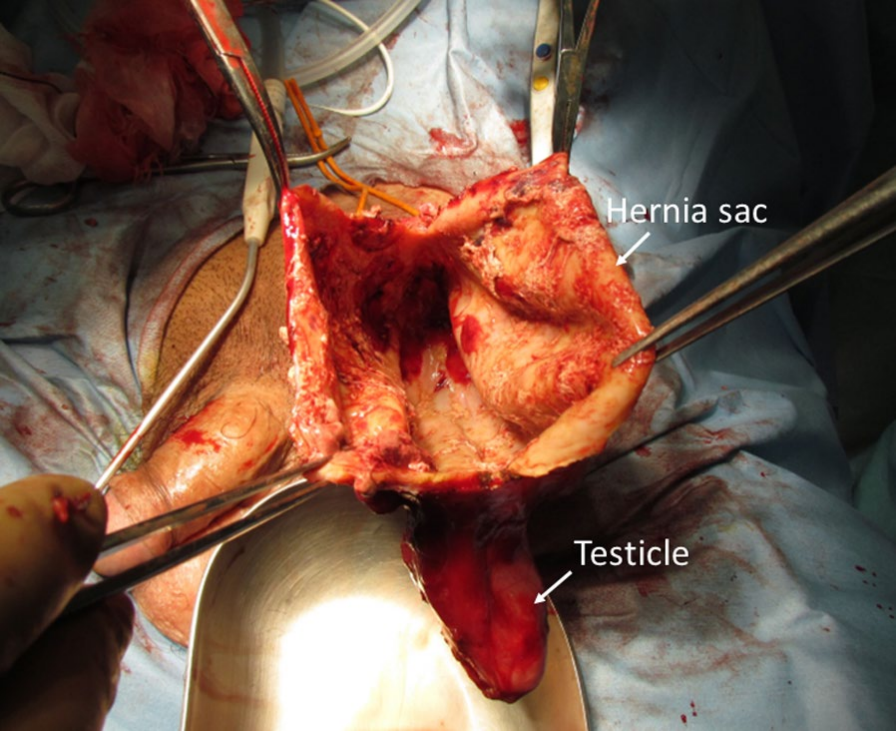
Intraoperative findings during the second operation. The walls of the hematocele were thickened

A hematocele 13 cm in diameter wrapped with the thickened hernia sac and tightly adherent to the spermatic cord and testicle was found (Figs. 4 and 5). The hematocele was removed from the scrotum with monopolar electrocautery, preserving the spermatic cord and testicle. There were no signs of inguinal hernia recurrence. The operative time was 113 min and the blood loss was 57 ml. A pathological study revealed a thickened hernia sac with an inflammatory cell infiltrate. The day after the surgery, scrotal swelling increased. Ultrasonography showed poor testicular perfusion and thus, an orchiectomy was performed on the third postoperative day. The resected testicle was filled with blood. Operative time was 62 min and blood loss was 69 ml. The postoperative course after the orchiectomy was uneventful and the patient was discharged on POD 7. The pathological finding was a hemorrhagic infarct of the testicle. No recurrence of inguinal hernia or scrotal swelling was observed at the 3-year follow-up.

## Discussion

The optimal procedure for GISH has not been established due to its rarity [2]; and two major problems, including recurrence and seroma, remain. For normal inguinal hernia, there was no difference in the recurrence rate between laparoscopic TAPP and the anterior approach [8]. Moreover, Cavalli et al. have documented the recurrence rate via anterior approach after GISH as high as 30% [9]; however, there have been no reports focused on recurrence of laparoscopic TAPP after GISH. We considered that laparoscopic TAPP had a great advantage in directly inspecting the hernia orifice followed by properly covering it with a prosthetic mesh (depending on the size of hernia orifice) over anterior approach [2, 6]. Hence, we regarded laparoscopic TAPP as an optimal procedure for GISH and usual inguinal hernia at our institution. In fact, we could successfully repair a large sized-hernia orifice by directly inspecting it and properly covering it with reinforcement mesh in the patient, and as a result, no recurrence was observed for 3 years after surgery. Therefore, in the present case, laparoscopic TAPP greatly contributed to successful repair for GISH.

The other major problem to be resolved is seroma following the hernia repair procedure, especially in laparoscopic TAPP. [10] Regarding normal inguinal hernia, several meta-analyses demonstrated that laparoscopic TAPP had a significantly higher incidence of seroma formation than that in open hernia repair [8, 11–14]. For GISH, Staubitz et al. have reported that only 1 out of 71 (1.4%) patients who underwent a herniorrhaphy with an anterior approach for GISH had a delayed scrotal hematocele, necessitating reoperation 6 months after the surgery, and 15 of 71 (21%) patients had postoperative scrotal fluid collections (seroma/hematocele) [15]. In contrast, there have only been four reports so far since 2011 [2, 5–7]. According to these reports, 7 out of 13 (53.8%) patients developed postoperative scrotal fluid collections after laparoscopic TAPP for GISHs. However, all patients except for one who required a puncture three times, recovered without an invasive procedure [2, 5–7]. These findings suggest that scrotal fluid collection was a recoverable complication, whereas an anterior approach is superior in preventing scrotal fluid collection to laparoscopic TAPP. However, in the present case, delayed refractory hematocele occurred, leading to reoperation after laparoscopic TAPP for a GISH. To the best of our knowledge, there have been no reports on the incidence of delayed scrotal fluid collection after laparoscopic TAPP for GISHs, and this is the first report about a severe complication leading to hemi-orchiectomy after laparoscopic TAPP for a GISH.

Three points are mainly considered as the causes of scrotal fluid collection after laparoscopic TAPP for GISH. The first is to leave plenty of empty space even after repair; second is to require a larger dissected area to lay the prosthetic mesh properly; and third is to have difficulty in fully removing the hernia sac due to technical limitations of the laparoscopic surgery. We believe that the main cause of such repercussions in laparoscopic TAPP for GISH was due to the presence of the residual hernia sac [16, 17]. Thus, the complete resection of the hernia sac plays an important role in reducing the incidence of postoperative scrotal fluid collection. On the other hand, it is controversial to remove the hernia sac in GISH. According to the EHS and IEHS guidelines, hernia sac transection and leaving the distal hernia sac undisturbed in GISHs are weakly recommended for preventing ischemic orchitis [3, 4]. Most patients with a GISH have a long disease history; therefore, there is often firm adhesion between the hernia sac and the testicle or the spermatic cord. In addition, a long-standing GISH can sometimes elongate the spermatic cord or the spermatic cord is twisted, causing testicular atrophy [18]. Therefore, total removal of a hernia sac has a potential risk of injury to the spermatic cord or orchitis [15]. Staubitz et al. reported that 14 of 71 (20%) patients who underwent herniorrhaphy with complete resection of the hernia sac via an anterior approach for GISH required intraoperative orchiectomy [15]. In the present case, the distal hernia sac was not completely resected to avoid spermatic cord injury. However, the remnant sac induced refractory hematocele and severe inflammation around the spermatic cord leading to firm adhesion. Further, dissection of the remnant hernia sac including the hematocele induced spermatic cord injury, leading to hemi-orchiectomy. Had we considered completely removing the giant hernia sac in the primary operation, orchiectomy might have been avoided in this case. Alternatively, had we carefully assessed the testis blood perfusion after dissecting the remnant sac from the spermatic cord in the secondary operation, at least the third operation could have been avoided.

To avoid this serious complication, we believe that it is important to perform total or subtotal hernia sac resection and that robotic procedures can contribute to this. Although there has been no established evidence that robotic surgery prevents the incidence of seroma after surgery when compared to laparoscopic surgery [19], we have previously demonstrated that the robotic system would be clinically more advantageous for more technically demanding procedures such as laparoscopic total gastrectomy [20]. Therefore, we hope to develop and establish a more feasible procedure to resect hernia sacs in GISHs using robotic systems in future. Until then, we consider it safer to perform the hernia repair via the anterior approach for GISH, despite its high recurrence rate.

## Conclusions

After performing laparoscopic TAPP for GISH, we encountered a case of a serious complication that required hemi-orchiectomy. When the hernia sac is not resected, we must be aware of the possibility that the remnant hernia sac will cause severe refractory hematoceles for GISHs. Furthermore, it should be taken into account that the removal of a hematocele from the spermatic cord and testicle might ultimately result in orchiectomy.

## Data Availability

Not applicable.

## Abbreviations

GISH: Giant inguinoscrotal hernia
EHS: European Hernia Society
IEHS: International Endohernia Society
TAPP: Transabdominal preperitoneal repair
POD: Postoperative day
